# Prognostic impact of Epstein-Barr virus (EBV)-DNA copy number at diagnosis in chronic lymphocytic leukemia

**DOI:** 10.18632/oncotarget.6281

**Published:** 2015-11-02

**Authors:** Jin-Hua Liang, Rui Gao, Yi Xia, Robert Peter Gale, Rui-Ze Chen, Yu-Qiong Yang, Li Wang, Xiao-Yan Qu, Hai-Rong Qiu, Lei Cao, Min Hong, Rong Wang, Yan Wang, Lei Fan, Yao-Yu Chen, Zhi-Bin Hu, Jian-Yong Li, Wei Xu

**Affiliations:** ^1^ Department of Hematology, The First Affiliated Hospital of Nanjing Medical University, Jiangsu Province Hospital, Nanjing, China; ^2^ Nanjing Medical University, Nanjing, China; ^3^ Haematology Research Centre, Division of Experimental Medicine, Department of Medicine, Imperial College London, London, United Kingdom; ^4^ Department of Epidemiology and Biostatistics, Nanjing Medical University, School of Public Health, Nanjing, China; ^5^ Collaborative Innovation Center for Cancer Personalized Medicine, Nanjing Medical University, Nanjing, China

**Keywords:** chronic lymphocytic leukemia, Epstein-Barr virus, prognosis

## Abstract

Epstein-Barr virus (EBV)-DNA is detected in the blood of some persons with chronic lymphocytic leukemia (CLL) at diagnosis. Whether this is important in the development or progression of CLL is controversial. We interrogated associations between blood EBV-DNA copy number and biological and clinical variables in 243 new-diagnosed consecutive subjects with CLL. Quantification of EBV-DNA copies was done by real-time quantitative PCR (RQ-PCR). All subjects had serological evidence of prior EBV-infection. However, only 24 subjects (10%) had a EBV-DNA-positive test at diagnosis. EBV-DNA-positive subjects at diagnosis had lower hemoglobin concentrations and platelet levels, higher thymidine kinase-1 and serum ferritin levels, un-mutated *IGHV* genes and a greater risk of Richter transformation compared with EBV-DNA-negative subjects. Percent CD20-, CD148- and ZAP70-positive cells and mean fluorescence intensity (MFI) of each cluster designation were also increased in EBV-DNA-positive subjects at diagnosis. EBV-DNA test positivity was associated with a briefer time-to-treatment interval (HR 1.85; [95% confidence interval, 1.13, 3.03]; *P*=0.014) and worse survival (HR 2.77; [1.18, 6.49]; *P=*0.019). Reduction in EBV copies was significantly associated with therapy-response. A positive blood EBV-DNA test at diagnosis and sequential testing of EBV copies during therapy were significantly associated with biological and clinical variables, time-to-treatment, therapy-response and survival. If validated these data may be added to CLL prognostic scoring systems.

## INTRODUCTION

EBV-infection is associated with several lymphomas such as Hodgkin lymphoma, Burkitt lymphoma, some T/NK-cell lymphomas and posttransplant lympho-proliferative disorders [1-3]. EBV can be detected in neoplastic B-cells in some persons with chronic lymphocytic leukemia (CLL) [4-8], especially those with Richter transformation [9-11]. However, associations between EBV-infection and CLL are controversial and an etiological role in CLL is still unproved [7-10]. Specifically, it is unclear if EBV-activation plays a role in causing CLL, whether the immune suppression intrinsic to CLL results in activation of latent EBV-infection, both or neither.

Studies of EBV-infection, typically re-activation of latent infection, in persons with CLL initially analyzed anti-EBV antibodies [12]. However, this approach is complex and unreliable because persons with CLL have impaired immunity and defective antibody responses to infections such as *Diplococcus pneumonia*, Herpes viruses like Varicella-zoster virus (VZV) and EBV [13, 14]. Recent studies directly quantify EBV-infection by measuring blood EBV-DNA [15]. Different compartments have been sampled using real-time quantitative polymerase chain reaction (RQ-PCR) including plasma, serum, mononuclear cells or whole blood [16]. EBV-DNA detected in these compartments by RT-PCR is associated with prognosis in several EBV-related lymphomas [17-22]. However, there are few if any similar studies in persons with CLL.

## RESULTS

### Subjects

Twenty four subjects (10%) had a positive EBV-DNA test at diagnosis. Median viral concentration was 6.30×10E+4 copies/mL (range, 5.20×10E+3 to 3.80×10E+5 copies/mL). Biological and clinical variables are compared between the EBV-DNA-positive and -negative cohorts in Table 1. All subjects had serological evidence of prior EBV-infection (EBNA-IgG-positive, EBV-CA-IgG-positive and/or EBV-CA-IgA-positive). Among the 24 subjects with EBV-DNA-positive, 21 had serological evidence of active EBV-infection (EBV-EA-IgG-positive). Significant differences included hemoglobin < 100g/L (*P* = 0.02), platelets < 100×10E+9/L (*P* = 0.04), thymidine kinase-1 >upper limits of normal (ULN; *P* < 0.01), serum ferritin (*P* = 0.03), immunoglobulin heavy-chain variable region (*IGHV*) un-mutated (*P* < 0.01) and Richter transformation (*P* = 0.03). Immunophenotypes are compared in Table 2. There was no significant association between EBV-DNA test result and *VH* family (Supplement Table 1) or *IGHV* gene usages (Supplement Table 2).

**Table 1:** Clinical and biological variables in EBV-DNA-positive and -negative subjects

Clinicalcharacteristics	EBV-DNA-positive	EBV-DNA-negative	*P-value*	Biologicalcharacteristics	EBV-DNA-positive	EBV-DNA-negative	*P-value*
Age >60 y	12/24	100/219	0.83	*TP53* disruption	4/22	39/200	1.00
Sex (Male)	18/24	151/219	0.65	*IGHV* un-mutated	15/24	70/219	**<0.01**
Rai ≥stage-2	13/24	109/219	0.83	Richter transformation	3/24	5/219	**0.03**
Binet ≥stage-B	11/24	127/219	0.28	-	-	-	-
ALC >50×10E+9/L	4/24	60/219	0.33	-	-	-	-
PLT <100×10E+9/L	10/24	49/219	**0.04**	-	-	-	-
HB <100 g/L	10/24	42/219	**0.02**	-	-	-	
SF >ULN	8/24	30/219	**0.03**	-	-	-	-
ALB <40 g/L	10/24	87/219	1.00	-	-	-	-
TK-1 >ULN	12/24	39/219	**<0.01**	-	-	-	-
LDH >ULN	8/24	56/219	0.47	-	-	-	-
β2-MG >ULN	20/24	151/219	0.17	-	-	-	-

Abbreviations: ALB: albumin; ALC: absolute lymphocytic counts; β2-MG: β2-microglobulin; HB: hemoglobine; *IGHV*: immunoglobulin heavy-chain variable region; LDH: lactate dehydrogenase; PLT: platelet; SF: serum ferritin; TK-1: thymidine kinase-1; ULN: upper limits of normal.

**Table 2:** The comparison of the expressions of the immunophenotypical markers between EBV-DNA-positive and EBV-DNA-negative CLL subjects

Markers	No. of cases	EBV-DNA-positive (mean, %)	EBV-DNA−negative (mean, %)	*P-*value	EBV-DNA-positive (mean, MFI)	EBV-DNA-negative (mean, MFI)	*P*-value
CD19	216	87%±2%	80%±1%	**0.029**	777±121	758±70	0.932
CD23	216	70%±4%	67%±1%	0.547	286±59	249±16	0.463
CD5	216	80%±3%	70%±1%	**0.020**	286±10	238±17	0.439
CD20	216	84%±2%	75%±1%	**0.022**	1209±254	799±66	0.064
CD22	216	21%±6%	20%±2%	0.833	31±4	27±4	0.744
CD200	214	75%±5%	77%±1%	0.652	248±46	233±14	0.741
CD148	214	78%±5%	71%±1%	0.073	280±45	197±14	0.075
CD38	210	20%±5%	17%±2%	0.557	105±76	36±7	**0.037**
ZAP70	210	31%±3%	21%±1%	**0.024**	69±26	32±6	**0.018**

### Impact of EBV-DNA testing and clinical outcomes

**Figure 1:**
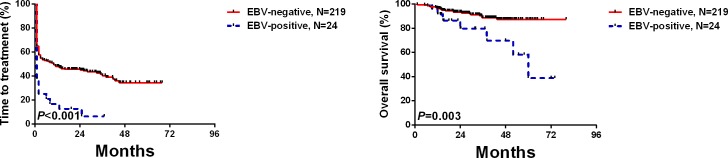
Freedom-from-therapy and survival by EBV-DNA test result at diagnosis

Median TTT was 6 months (range, 1−71 months). Median survival was 74 months (range, 2−83 months). 3-year survival was 89% (95% confidence interval [CI], 87, 91%). At two months after diagnosis 18 subjects in the EBV-DNA-positive cohort were treated *vs.* 93 of 219 subjects (42% in the EBV-DNA-negative cohort (*P* < 0.001). Median survival was 60 months (range, 9−74 months) in the EBV-DNA-positive cohort *vs.* not reached (*P* < 0.001; Figure 1) in the EBV-DNA-negative cohort.

Variables significantly associated with TTT interval in univariate analyses were entered into multivariate analyses. Five parameters, Binet ≥stage-B (HR, 1.45; [1.01, 2.09]; *P* = 0.044), lymphocytes >50×10E+9/L (HR, 1.41 [1.12, 2.32]; *P* = 0.010), EBV-DNA positivity (HR, 1.85 [1.13, 3.03]; *P* = 0.014), un-mutated *IGHV* state (HR, 1.87 [1.28, 2.71]; *P* = 0.001) and albumin < 40 g/L (HR, 1.17 [1.09, 2.26]; *P* = 0.015) were significantly associated with TTT interval (Table 3). Variables significantly associated with survival in univariate analyses were entered into multivariate analyses. EBV-DNA-positivity (HR, 2.77 [1.18, 6.49]; *P* = 0.019), *TP53* disruption (HR, 1.75 [1.04, 5.50]; *P* = 0.040) and un-mutated *IGHV* state (HR, 2.67 [1.04, 6.88]; *P* = 0.042) were significantly associated with survival (Table 4).

**Table 3:** Univariate and multivariate Cox regression analysis of TTT for CLL subjects (*N*=243)

	Univariate analysis			Multivariate analysis		
Characteristics	HR	95%CI	*P-*value	HR	95%CI	*P-*value
Age >60 y	1.00	0.73-1.38	0.985	-	-	-
Sex (male)	0.99	0.70-1.39	0.935	-	-	-
Binet ≥stage-B	1.53	1.01-2.14	**0.012**	1.45	1.01-2.09	**0.044**
ALC >50×10E+9/L	1.52	1.18-2.74	**0.016**	1.41	1.12-2.32	**0.010**
ALB <40g/L	1.39	1.01-1.92	**0.043**	1.17	1.09-2.26	**0.015**
LDH >ULN	1.90	1.36-2.67	**<0.001**	1.28	0.87-1.89	0.215
β2-MG >ULN	1.60	1.09-2.33	**0.016**	1.12	0.73-1.71	0.615
EBV-DNA positivity	2.08	1.32-3.29	**0.002**	1.85	1.13-3.03	**0.014**
*TP53* disruption	1.64	1.12-2.40	**0.011**	1.11	0.72-1.71	0.631
*IGHV* unmutated	2.15	1.55-2.98	**<0.001**	1.87	1.28-2.71	**0.001**
PLT <100×10E+9/L	1.38	0.96-1.98	0.081	-	-	-
HB <100 g/L	1.26	0.86-1.84	0.231	-	-	-
SF >ULN	1.18	0.78-1.78	0.443	-	-	-
TK-1 >ULN	1.09	0.74-1.61	0.662	-	-	-
CD38 (≥30%)	1.26	0.88-1.81	0.214	-	-	-
ZAP70 (≥20%)	1.01	0.72-1.41	0.969	-	-	-

Abbreviations: ALB: albumin; ALC: absolute lymphocytic counts; β2-MG: β2-microglobulin; HB: hemoglobine; *IGHV*: immunoglobulin heavy-chain variable region; LDH: lactate dehydrogenase; PLT: platelet; SF: serum ferritin; TK-1: thymidine kinase-1; ULN: upper limits of normal.

**Table 4:** Univariate and multivariate Cox regression analysis of OS for CLL

	Univariate analysis			Multivariate analysis		
Characteristic	HR	95%CI	*P-*value	HR	95%CI	*P-*value
Age >60 y	2.02	0.98-4.16	0.059			
Sex (male)	1.24	0.55-2.81	0.603			
Binet ≥stage-B	2.28	1.02-5.11	**0.045**	1.33	0.54-3.27	0.541
ALC >50×10E+9/L	1.02	0.45-2.31	0.957			
ALB <40g/L	1.64	0.79-3.44	0.188			
LDH >ULN	3.19	1.55-6.56	0.002	1.75	0.74-4.12	0.199
β2-MG >ULN	1.75	0.72-4.28	0.220			
EBV-DNA positivity	5.20	2.43-11.12	**<0.001**	2.77	1.18-6.49	**0.019**
*TP53* disruption	4.06	1.95-8.47	**<0.001**	1.75	1.04-5.50	**0.040**
*IGHV* unmutated	5.06	2.32-11.01	**<0.001**	2.67	1.04-6.88	**0.042**
PLT <100×10E+9/L	1.13	0.52-2.46	0.768	-	-	-
HB <100 g/L	1.28	0.59-2.80	0.536	-	-	-
SF >ULN	2.04	0.94-4.45	0.073	-	-	-
TK-1 >ULN	1.74	0.82-3.73	0.152	-	-	-
CD38 (≥30%)	2.30	1.11-4.74	**0.023**	1.61	0.73-3.55	0.237
ZAP70 (≥20%)	1.28	0.60-2.73	0.518	-	-	-

Abbreviations: ALB: albumin; ALC: absolute lymphocytic counts; β2-MG: β2-microglobulin; HB: hemoglobine; *IGHV*: immunoglobulin heavy-chain variable region; LDH: lactate dehydrogenase; PLT: platelet; SF: serum ferritin; TK-1: thymidine kinase-1; ULN: upper limits of normal.

### Changes in EBV copies and therapy-response

**Figure 2:**
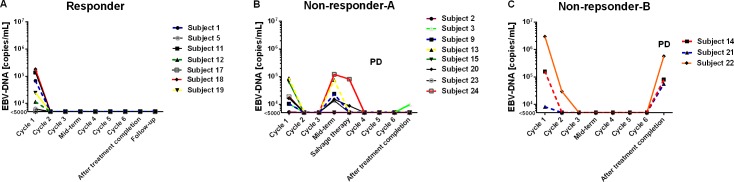
EBV-DNA copies and therapy-response

Eighteen subjects with an EBV-DNA-positive test at diagnosis had sequential measurements of EBV copies during therapy and follow-up (Figure 2). Seven patients of therapy responders had a significant reduction of EBV-DNA copies (Figure 2A). In contrast, 6 subjects failing therapy had a rapid increase in EBV-DNA copy numbers (Figure 2B). Three subjects had increases in EBV-DNA copies when they developed Richter transformation (Figure 2C).

### EBV-DNA copy number and WBC

Because EBV genomes are mostly intra-cellular, we interrogated whether viral load in whole blood was associated with WBC levels. There was no correlation between WBC levels and EBV-DNA-test result (positive: median, 27×10E+9/L; range, 2−175×10E+9/L *vs.* negative: median, 25×10E+9/L; range, 2−252×10E+9/L; *P* = 0.51). Also, in the 24 subjects with a positive EBV-DNA test there was no significant association between the EBV-DNA copy number and WBC level (*r* = 0.253, *P* = 0.244). Finally, EBV-DNA copy number in 1×10E+6 cells blood mononuclear cells from five EBV-DNA positive subjects varied substantially. These data indicate the association between a positive EBV-DNA test and biological and clinical variables is independent of WBC level.

## DISCUSSION

A positive EBV-DNA test at diagnosis was significantly associated with several clinical and biological variables previously reported to correlate with a poor prognosis in persons with CLL. However, the adverse impact of a positive EBV-DNA test persisted as an independent predictor of TTT and survival in multivariate analyses. These data suggest an important role for EBV-infection and/or reactivation in some persons with CLL.

The 10% incidence of EBV-DNA we detected in whole blood at diagnosis is similar to 14% reported using a EBV-encoded latent membrane protein 1 (EBV LMP-1) mRNA transcript test reported by Tarrand JJ *et al.* [6] but less than the 38% detected by *in situ* hybridization for EBV-encoded small RNA1 (EBV-EBER1) reported by Tsimberidou AM *et al*. [4]. This difference is likely because EBER-1 is can be detected in latent EBV-infection whereas EBV-DNA and EBV-LMP1 mRNA are found only during active infection [4]. The different test sensitivities of FISH and RT-PCR might be also the reason for the difference. The associations we report between a positive EBV-DNA blood test and worse outcomes are similar to those reported using a test for EBV-EBER1 and of LMP1 mRNA [4, 6, 10]. EBV-infection stimulates proliferation of B-cells and inhibits apoptosis. Changes in immunophenotype are consistent with this notion and suggest EBV-infection, re-activation and/or -replication may be important in the biological and clinical features of CLL in some persons.

Presence of EBV-EBER1 and EBV-LMP-1 mRNA in persons with CLL are associated with briefer TTT, increased risk of Richter transformation and worse survival in several studies [4, 10, 23, 24]. We also found EBV-DNA-positive subjects were also more likely to have Richter transformation. Three subjects who were EBV-DNA-positive at diagnosis and who became -negative with therapy had an increase in EBV copy numbers at the time of Richter transformation. These data suggest an association between EBV-infection, re-activation and/or -replication and Richter transformation.

The explanation for the association between EBV-DNA and-infection and/or re-activation and Richter transformation is complex. EBV-infection and/or reactivation might cause Richter transformation as it does Burkitt lymphoma. Alternatively, development of Richter syndrome might activate EBV-infection. These interactions are not mutually-exclusive and one, both or neither might operate in different persons.

Similar to the study reported by Visco C et al. [25], the etiological role of EBV in CLL cannot be defined in the present study. However, there are several potential explanations of why patients with high EBV DNA loads had poorer outcomes. First, EBV replication causes severe immune suppression. Consequently, persons with a high EBV-DNA load are at increased infection-risk and may have been less able to tolerate anti-CLL therapy. Second, Terrin L et al. [26] reported latently-EBV-infected B-cells induced sustained telomerase which was associated with an aggressive clinical behavior [27]. Third, we observed an association between high EBV-DNA load and un-mutated *IGHV* which was independently-associated with poorer survivals.

Our data indicate an association between blood EBV-DNA test positivity, EBV copy numbers and therapy-response. Because we did not give specific anti-EBV therapy, because drugs used to treat CLL lack anti-EBV activity and because we found no correlation between WBC level and EBV-DNA copy number, we assume this association reflects a biological property of the CLL cells, the host or both. Similar associations are reported in subjects with Hodgkin lymphoma, extra-nodal NK/T-cell lymphoma, diffuse large B-cell lymphoma and nasopharyngeal carcinoma [14, 20-22, 28-31]. These data are consistent with a correlation between EBV-DNA copy levels and disease activity and, if validated, could be used to predict outcomes.

## MATERIALS AND METHODS

### Subjects

Two hundred and forty-three newly diagnosed CLL persons were enrolled in this study from February 2008 to January 2014. Diagnosis of CLL was based on criteria of the International Workshop on CLL-National Cancer Institute (IWCLL-NCI) [32, 33]. The study was approved by the Ethics Committee of the First Affiliated Hospital of Nanjing Medical University. Subjects provided written informed consent and carried out according to the Declaration of Helsinki.

Baseline clinical and biological variables tested are summarized in Table 1. Anti-EBV antibody tests (EBNA-IgG, EBV-CA-IgG, EBV-CA-IgA, EBV-CA-IgM and EBV-EA-IgG) were done in all subjects. immunophenotype analyses included CD5, CD19, CD23, CD20, CD22, CD148, CD200, CD38 and ZAP70 measured by flow cytometry (Table 2). Results for each cluster designation and ZAP70 were expressed as percent positive cells and as mean fluorescence intensity (MFI). Methods and cut-off values are previously reported [34-36].

Subjects received therapy according to recommended guidelines [32, 33]. One hundred and fifty-one subjects (62%) received induction therapy with fludarabine, cyclophosphamide and rituximab (*N* = 69; 46%, fludarabine and cyclophosphamide (*N* = 47; 31%), bendamustine (*N* = 21; 14%) and chlorambucil (*N* = 14; 9%). Physicians were blinded to results of EBV-DNA tests when selecting therapy.

### EBV-DNA by real-time PCR

Whole blood samples were collected in an EDTA-containing tube and DNA extracted with the EBV-PCR Fluorescence Quantitative Diagnostic Kit (Da An Gene Co., Guangzhou, China). Quantification of EBV-specific sequences was performed by RQ-PCR assay with an ABI PRISM 7500 (Applied Biosystems, Foster City, CA, USA). Copy number was calculated from a standard curve. The lower boundary of test sensitivity was 5×10E+3 copies/mL [22]. Subjects with values < 5×10E+3 copies/mL were scored as EBV-DNA-negative including potentially subjects with no copies and subjects with values of 1 to < 5×10E+3 copies/mL.

### Statistics

Fisher exact test was applied to categorical variables and the Mann-Whitney U test to continuous variables. Time-to-treatment (TTT) interval was defined as interval from diagnosis to first treatment. Survival was defined as interval from diagnosis to death, loss to follow-up or May, 2015. Survival curves were constructed by Kaplan-Meier method, and log-rank test used to test for significant differences. Associations between variables and EBV-DNA testing results were interrogated in univariate analyses. Variables with significant associations were included in multivariate Cox proportional hazards regression analyses. Statistical analyses were performed using SPSS software for Windows (version, 17.0). *P*-values < 0.05 were considered significant. Data were analyzed as of 1-Jan-2015. Median follow-up is 41 months (range, 14-83 months).

## SUPPLEMENTARY MATERIAL TABLES


