# Supplementary material for: Prognostic impact of Epstein-Barr virus (EBV)-DNA copy number at diagnosis in chronic lymphocytic leukemia
Source: Oncotarget. 2015 Nov 2;7(2):2135–42. doi: 10.18632/oncotarget.6281 (PMC4811522; doi:10.18632/oncotarget.6281)
Supplement: Supplementary file 1 [file oncotarget-07-2135-s001.pdf]

Prognostic impact of Epstein-Barr Virus (EBV)-DNA copy number at diagnosis in chronic lymphocytic leukemia

Supplementary Material

Supplement Table 1: Correlation of EBV infection and VH family

	EBV-DNA-positive (N)	EBV-DNA-negative (N)	*P*-value
**VH-1**			0.286
Yes	4	22	
No	17	177	
**VH-2**			1.000
Yes	0	6	
No	21	193	
**VH-3**			0.357
Yes	9	111	
No	12	88	
**VH-4**			0.174
Yes	8	51	
No	11	148	
**VH-5**			1.000
Yes	0	3	
No	21	196	

Supplement Table 2: The correlation of EBV infection and 7 mostly used IGHV genes in the 243 CLL subjects in China

	EBV-DNA-positive (N)	EBV-DNA-negative (N)	*P*-value
**VH4-34**			0.202
Yes	5	27	
No	16	172	
**VH3-23**			0.449
Yes	3	19	
No	18	180	
**VH3-7**			1.000
Yes	2	21	
No	19	178	
**VH4-39**			1.000
Yes	1	8	
No	20	181	
**VH1-69**			0.397
Yes	1	4	
No	20	195	
**VH4-59**			0.581
Yes	1	7	
No	20	192	
**VH3-21**			1.000
Yes	0	4	
No	21	195	
